# Progress in Surgery

**Published:** 1901-12-07

**Authors:** 


### Progress in Surgery.

GENITO-URINARY—*(Continued from page 153).*

**Urethroscopy.**—Wm. K. Otis (New York) has given an account of a new form of urethroscope.[4] He alludes to the two distinct methods of illuminating the urethra—that in which the electric lamp is introduced into the urethroscopic tube, and that in which the light rays are thrown down the tube from an external lamp by means of a mirror, prism, or lens. Objections to the former method are : that a portion of the field is obstructed by the lamp, that light carriers of various lengths corresponding to the length of each tube which is used must be employed, that the tiny lamp is usually short lived, and, more important than the above, it is practically impossible to render the instrument perfectly clean, and the presence of the lamp hinders the use of instruments passed along the tube. The lamp frequently gets covered with blood or pus. The Otis urethroscope consists of a short metal tube bent at a right angle about its middle, which carries the light and a lens in front which directs the light to the bottom of the urethroscopic tube. The handle bearing the conducting wires fits into the vertical part of the metal tube described above, and has a switch attached. A strong wire connects the instrument to the urethroscopic tube. The illuminating apparatus stands below the level of the urethroscopic tube so that there is no interference with a direct view of the field or with the introduction of instruments.

**Immediate Suture of the Bladder after Suprapubic Cystotomy.**—Bălăcescu (Bukarest)[5] advocates this procedure and describes the technique of a method which has proved very successful. He says there is no contraindication to immediate suture except hæmorrhage from the bladder ; and that experience shows cystitis is not an absolute contraindication. Immediate suture is getting more and more popular. The bladder wound heals in seven days and the patient can leave his bed in twelve days and return to work. The patient is prepared beforehand by having the bladder washed out with 1 in 2,000 permanganate of potash solution or 1 in 5,000 nitrate of silver solution, and is given salol or urotropine internally. Three to six days are devoted to this preparation. The bladder is completely emptied for the operation. Its wall is easy to recognise. The peritoneum is slightly separated from the bladder above. The mode of suture of the bladder wound, the so-called *Cystorrhaphie per Imbrikation*, is rather complicated. First from the left side of the wound a crescentic portion of mucous membrane is peeled off the muscular layers and removed. The free edge of mucous membrane thus formed is then sutured to the edge of the mucous membrane of the right side which has not been separated from the muscular layers, with a continuous suture. The muscular flap, denuded of mucous membrane, which remains on the left side is then fixed at its base to the edge of the muscular coat on the right side by a second continuous suture. Finally, with a third suture the rest of the muscular flap which remains free is fixed on to the surface of the muscle on the opposite side. Twelve cases are tabulated. Primary union resulted in eleven of these, and slight urinary infiltration in one. Four of the cases were allowed to urinate naturally from the beginning, and this is the plan the various operators mentioned favour. Loumeau[6] states that in the case of the bladder, as in all other operations, suturing is the method of choice. He relates the case of a man aged 80 years who had had cystitis and three large phosphatic calculi, and whose bladder was closed with a double row of sutures after the stones had been removed suprapubically. The man made an uninterrupted recovery, except for transient phlebitis in one leg, and returned home in three weeks. Edgar Huntley[7] publishes another successful case, that of a boy six years of age with stone in the bladder. Nine Lambert's sutures were used to close the bladder. A catheter was tied in the urethra for five days.

**Tumours of the Bladder.**—Wm. H. Battle[8] records a case of tumour involving the urethra and neck of the bladder. The patient was a woman, 58 years of age, who had been losing weight and strength for three months, but had only had pain and frequency of micturition about six weeks. The growth was a hard ovoid mass the size of a chestnut and extended from the meatus, which it surrounded, back to the bladder. It was somewhat fixed to the pubes, and was palpable through, but not involving, the vaginal mucous membrane. Suprapubic cystotomy was first performed and the bladder wall stitched to the skin. Then, with the patient in the lithotomy position, the growth was excised through the anterior vaginal wall. Thus the neck of the bladder was removed. The resulting wound in the bladder was closed with sutures. Slight leakage occurred once or twice through the wound, but finally it closed completely. The patient was watched for nine months. She wore a suprapubic tube with comfort, and gained flesh. The growth was a papilliferous columnar-celled carcinoma. Battle refers to another case of the same kind he published in 1895. Another is mentioned which was done by J. H. Dunn (U.S.A.) with great relief to the patient. These growths, in contrast to caruncles, only excite pain late in their evolution. David Wallace,[9] discussing bladder tumours, mentioned a case in which a tumour the size of a filbert in a woman of 60 almost killed the patient with the severity of the hæmorrhage, whereas in another case, a man of 23, the bladder was almost filled with papillomatous growths and yet the bleeding was never serious. Examination of the urine for tumour cells usually gives negative results, but if large nucleated cells are seen in groups the diagnosis of tumour is assisted, though the cells may not show the nature of the growth. Speaking on the prognosis in these cases Wallace points out that a tumour with a narrow pedicle may have deep attachments and may invade the mucous membrane some distance beyond the apparent attachment. In two cases he has seen rapid recurrence after removal of pedunculated growths. The cessation of hæmorrhage after suprapubic cystotomy is often remarkable. It is stated that the bladder may be kept quite empty and the patient perfectly dry with Cathcart's adaptation of the Sprengel pump. The writer of this abstract has not found that apparatus so perfect in these respects as stated above. Loewenhardt (Breslau) [10] has written on the intra-vesical methods of dealing with bladder tumours, with the modern operating cystoscopes. He alludes to the scanty records published of cases so dealt with, and the objections surgeons seem to have to the procedures. Previous exercise on the phantom bladder is necessary to be successful in manipulations with these instruments. In the newest Nitze operating cystoscopes the snare, cautery and washing-out appliances are combined in one instrument. It is alleged that a better view of the bladder is obtained with a cystoscope, the bladder being distended, than after suprapubic cystotomy when the bladder becomes collapsed. In illustration of this the writer mentions a case in which a small tumour was overlooked when the bladder was opened, and was subsequently seen and removed with the cystoscope. Kelly's method of conducting operations on the female bladder with a speculum passed through the urethra is considered to be no longer defensible, and dilatation of the urethra to be superfluous. The same applies to colpocystotomy for small papillomas of the bladder. Advantages of the cystoscopic method which will insure its more extensive use, are that a general anæsthetic is dispensed with, the patient is confined to bed a very short time, and the very small risk attendant on the procedures. Loewenhardt has found no recurrence in six cases of papilloma removed with the instrument. It is hoped that the calibre of the instrument may in the future be still further diminished, as its use is liable to be hindered at present by the pressure it exerts on the urethra and the neck of the bladder.

**The Sterilisation of Catheters.**—M. W. Herman [11] states that a safe and convenient method of sterilisation which is suitable for all kinds of catheters has not hitherto been described. Metal and Nélaton (French) catheters can be sterilised by simple boiling, but silk catheters become rough and cracked if so treated. He states that the latter may be boiled in a saturated solution of sulphate of ammonia withont being injured in any way. After five hours' continuous boiling in this solution these catheters retained all their polish, and also they bore equally well repeated boiling after intervals of use. After being boiled they may be lubricated with vaseline and used immediately, as the fluid does not irritate the urethra. Infected catheters may thus be sterilised by boiling in three to five minutes. The same fluid may be used for sterilising Nélaton and elastic catheters. No other method described up to the present time seems so simple and effectual as this.

[4] Lancet, Aug. 31, 1901, p. 599. [5] Centralb. f. Chir., No. 25, June 22, p. 633. [6] Vide Abst. Amer. Jour. Med. Sciences, July 1901, p. 101. [7] Lancet, May 11, 1901, p. 1333. [8] Lancet. July 13, 1901, p. 79. [9] Ibid, p. 65. [10] Beilage z. Centralb. f. Chir., No. 29, p. 100. [11] Vide Abst. Brit. Med. Jour. Epitome, June 1, 1901.

